# Intravenous injection of cyclophilin A realizes the transient and reversible opening of barrier of neural vasculature through basigin in endothelial cells

**DOI:** 10.1038/s41598-021-98163-w

**Published:** 2021-09-29

**Authors:** Narumi Nakada-Honda, Dan Cui, Satoshi Matsuda, Eiji Ikeda

**Affiliations:** 1 Department of Pathology, Yamaguchi University Graduate School of Medicine, 1-1-1 Minami-Kogushi, Ube, Yamaguchi 755-8505 Japan; 2 Department of Cell Signaling, Institute of Biomedical Sciences, Kansai Medical University, 2-5-1 Shinmachi, Hirakata, Osaka 573-1010 Japan

**Keywords:** Blood-brain barrier, Cardiovascular biology

## Abstract

Neural vasculature forms the blood–brain barrier against the delivery of systemically administered therapeutic drugs into parenchyma of neural tissues. Therefore, procedures to open the blood–brain barrier with minimal damage to tissues would lead to the great progress in therapeutic strategy for intractable neural diseases. In this study, through analyses with mouse in vitro brain microvascular endothelial cells and in vivo neural vasculature, we demonstrate that the administration of cyclophilin A (CypA), a ligand of basigin which is expressed in barrier-forming endothelial cells, realizes the artificial opening of blood–brain barrier. Monolayers of endothelial cells lost their barrier properties through the disappearance of claudin-5, an integral tight junction molecule, from cell membranes in a transient and reversible manner. Furthermore, the intravenous injection of a single dose of CypA into mice resulted in the opening of blood–brain barrier for a certain period which enabled the enhanced delivery of systemically administered doxorubicin into the parenchyma of neural tissues. These findings that the pre-injection of a single dose of CypA realizes an artificial, transient as well as reversible opening of blood–brain barrier are considered to be a great step toward the establishment of therapeutic protocols to overcome the intractability of neural diseases.

## Introduction

Movement of molecules from blood to parenchyma of neural tissues is strictly restricted by the blood–brain barrier function of neural vasculature. Multicellular organisms have acquired this vascular barrier function to maintain the optimal microenvironment of neural tissues in which neural cells can function normally^1,2^. However, during the progress in medicine, the blood–brain barrier has shown another aspect of a barrier against the delivery of systemically administered therapeutic drugs into cerebral parenchyma affected in neural diseases^2,3^. In the research of drug discovery for neural diseases, the difficulty in drug delivery into cerebral parenchyma is one of the most important problems to be overcome, and the establishment of procedures to allow drugs to pass through the blood–brain barrier artificially as well as appropriately would lead to the revolutionary therapies with a wide range of application for various intractable neural diseases.

Previously, we demonstrated that the subcellular localization of claudin-5, which is one of the integral molecules for tight junction assembly^4^, to endothelial cell membranes is the critical phenomenon and the index of blood–brain barrier function^5^. Subsequently, we specified three transmembrane molecules, a disintegrin and metalloprotainases (ADAMs) 12 and 17 as well as basigin, as the molecules involved in the disappearance of claudin-5 from endothelial cell membranes and the consequent loss of blood–brain barrier function under pathological stimuli such as tissue hypoxia, inflammatory cytokines and so forth^6,7^. Among these molecules, basigin was shown to be essential commonly for the loss of blood–brain barrier function by various pathological stimuli, in contrast to the involvement of ADAM12 and ADAM17 predominantly in hypoxia-induced loss of barrier function^7^. Therefore, taking the clinical application into consideration, we have focused our studies on basigin to establish the procedures for artificial regulation of blood–brain barrier function. As for the restoration of pathologically impaired barrier function, we reported, with the model of diabetic retinopathy, the availability of inhibitory reagents against basigin^7^.

In the present study, we have attempted to establish the procedure for artificial opening of blood–brain barrier by using basigin as a target molecule. Basigin is known to be a receptor for cyclophilin A (CypA), a member of cyclophilins with peptidyl-prolyl cis–trans isomerase (PPIase) activity. CypA was originally identified as an intracellular receptor for the immunosuppressive drug cyclosporin A (CsA), and the formation of CypA-CsA complex results in the inhibition of calcineurin which prevents T cell activation^8^. Accumulating evidence has disclosed the critical involvement of intracellular CypA in various cellular functions such as cell signaling, protein folding, protein trafficking and transcriptional regulation. Recently, in addition to the intracellular CypA, the important role of secreted CypA in diverse physiological as well as pathological situations has been highlighted. CypA is reported to be secreted by various cell types such as vascular smooth muscle cells, endothelial cells and macrophages, and the extracellular CypA modulates the pathological processes including those in inflammatory as well as cardiovascular diseases through binding to basigin at the surface of target cells^8–10^. Here, we demonstrate that the extracellular CypA acts directly on neural vascular endothelial cells to weaken their barrier properties, and furthermore that the intravenous injection of a single dose of CypA realizes the transient as well as reversible opening of blood–brain barrier with a period sufficient enough to allow the systemically administered therapeutic drugs to reach the parenchyma of neural tissues.

## Results

### Effect of CypA on barrier properties of brain microvascular endothelial cells

**Figure 1:**
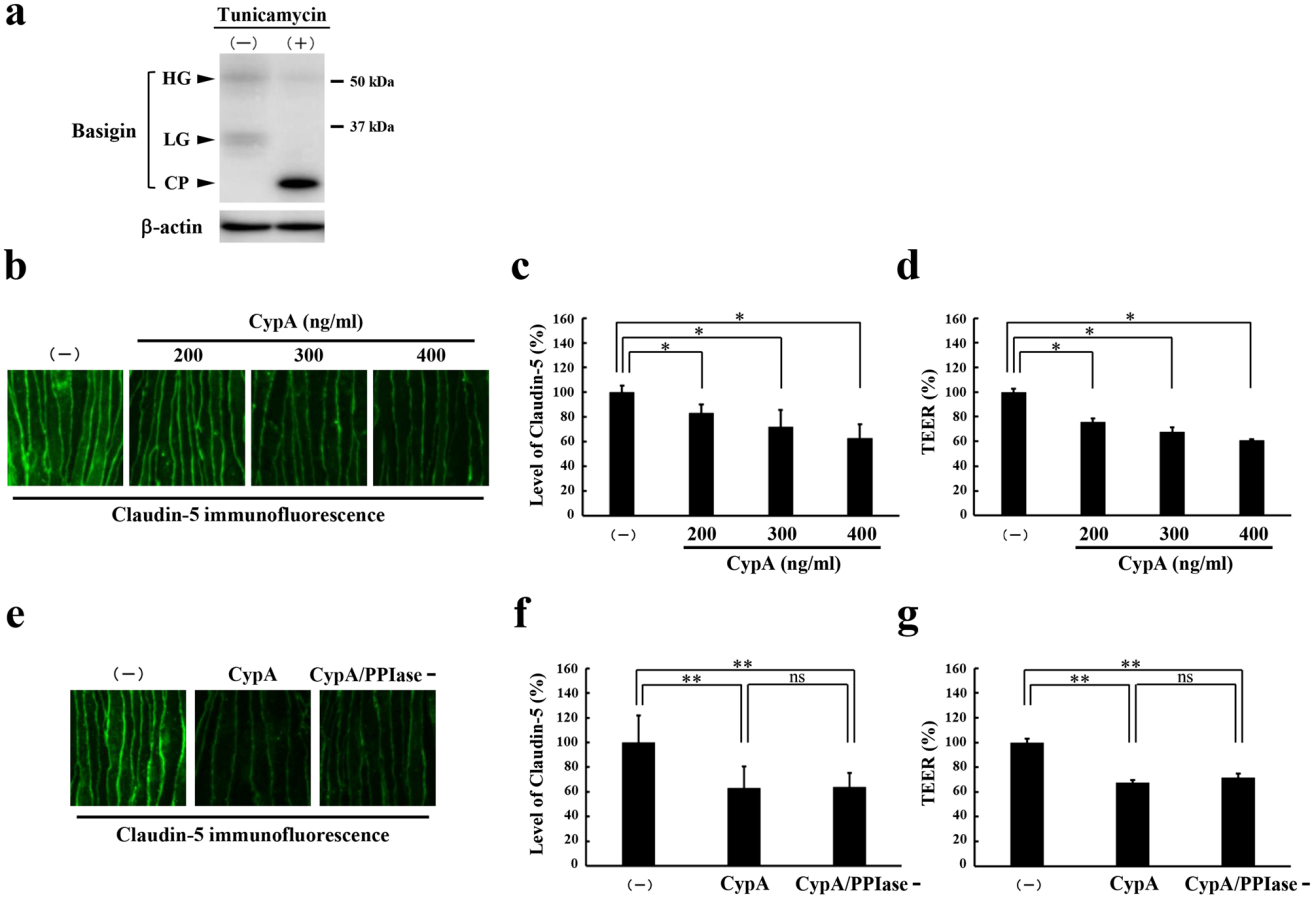
Effect of CypA on the expression of claudin-5 in brain microvascular endothelial cells. (**a**) bEnd.3 monolayers express basigin in two different glycosylation forms. Full-length blots are presented in Supplementary Fig. S1. (**b**–**d**) Monolayers were stimulated by CypA at concentration of 200, 300 or 400 ng/ml, and the level of cell membrane-localized claudin-5 (**b**,**c**) as well as the barrier property (**d**) were determined by immunofluorescence staining (**b**) with its corresponding quantitative analysis (**c**) and TEER (**d**), respectively. Significant decrease in the levels of cell membrane-localized claudin-5 are detected in monolayers treated with CypA at all the concentrations examined in a close correlation with the decrease in TEERs of monolayers. (**e**–**g**) Monolayers were treated with CypA or CypA/PPIase- which lacks PPIase activity. Immunofluorescence staining (**e**) with its corresponding quantitative analysis (**f**) as well as the measurement of TEER (**g**) indicate that CypA-induced decrease in cell membrane-localized claudin-5 is independent of PPIase activity. Data in (**c**,**d**,**f**,**g**) are presented as mean ± SD from 3 independent experiments. *HG* high glycosylation form, *LG* low glycosylation form, *CP* core protein. *P < 0.05; **P < 0.01; *ns* not significant.

**Figure 2:**
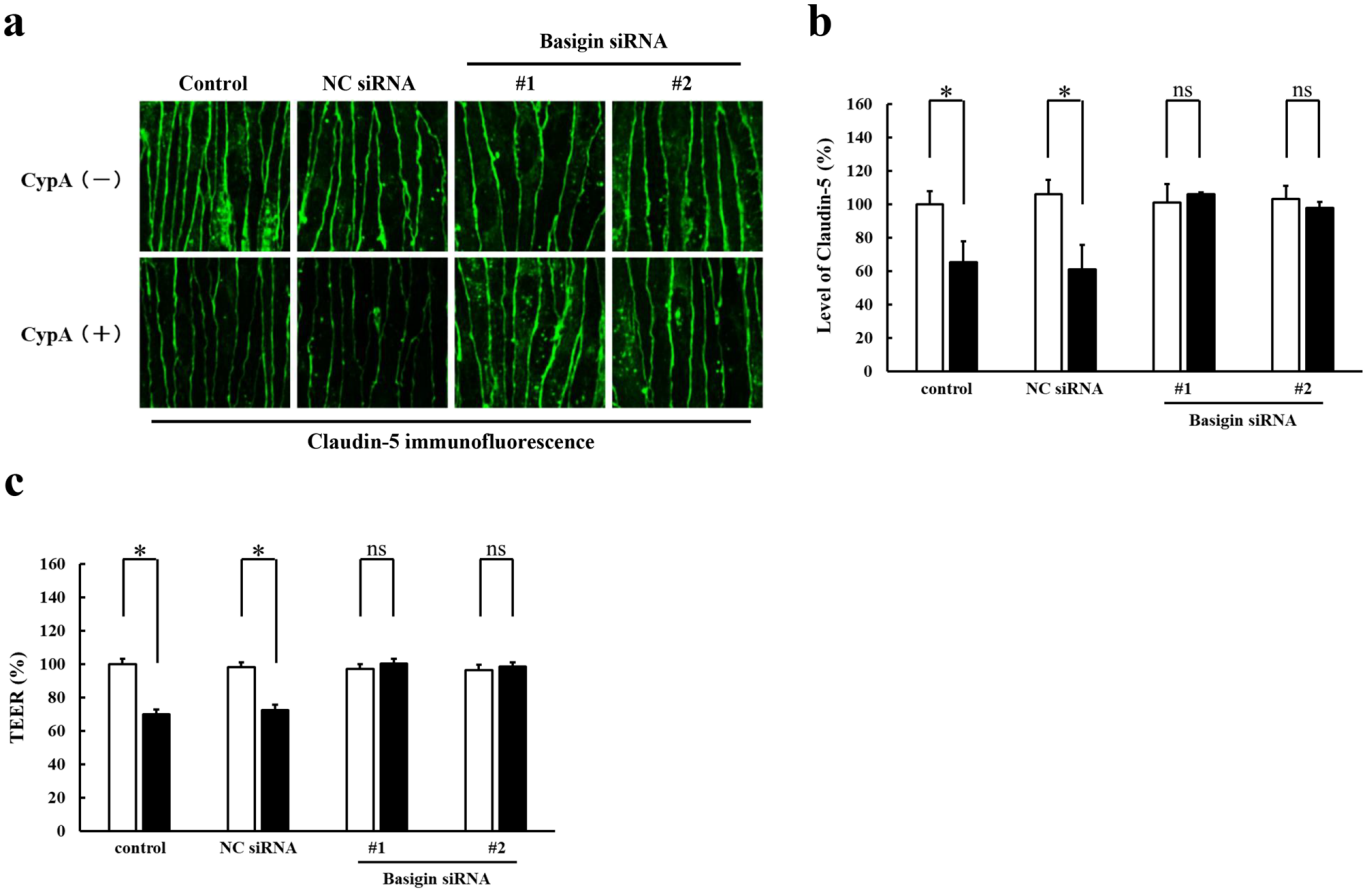
Involvement of basigin in CypA-induced disappearance of claudin-5 from cell membranes of brain microvascular endothelial cells. Immunofluorescence staining (**a**) with its corresponding quantitative analysis (**b**) as well as the measurement of TEER (**c**) demonstrate that CypA-induced disappearance of claudin-5 from cell membranes and the decrease in TEER of bEnd.3 monolayers are inhibited by the suppression of basigin expression with specific siRNAs (#1 and #2). *NC siRNA* non-silencing siRNA for negative control. *P < 0.01; *ns* not significant.

For an in vitro model of blood–brain barrier, a mouse brain microvascular endothelial cell line, bEnd.3, was cultured at the confluent state for 7 days to obtain the monolayer of endothelial cells with the claudin-5 expression exclusively in cell membranes and the consequent increase in transendothelial electrical resistance (TEER), an index of barrier function, as described previously^5–7,11^. First, the expression of basigin in bEnd.3 monolayers was examined by Western blot analysis. Basigin was shown to be expressed in two different forms which were confirmed by an analysis with tunicamycin to correspond to the high glycosylation and low glycosylation forms of basigin (Fig. 1a, Supplementary Fig. S1). Then, bEnd.3 monolayers were cultured in the absence or presence of CypA of different concentrations. Levels of cell membrane-localized claudin-5 and the barrier function of monolayers were monitored by immunostaining with quantitative analyses and measurement of TEER, respectively^6,7^. Either 200, 300 or 400 ng/ml of CypA treatment for 3 h was shown to decrease the levels of cell membrane-localized claudin-5 as well as the TEERs of monolayers, indicating that CypA treatment results in the loss of barrier properties of bEnd.3 cells (Fig. 1b–d). According to the studies reported so far, some biological phenomena triggered by CypA depend on its PPIase activity, while others do not require PPIase activity^10,12,13^. Therefore, it was interesting to know if PPIase activity is involved in the loss of barrier properties of bEnd.3 monolayers by CypA treatment. To answer this question, we treated bEnd.3 monolayers with CypA lacking PPIase activity (CypA/PPIase−). Treatment with CypA/PPIase− was shown to decrease the levels of cell membrane-localized claudin-5 as well as the TEERs of monolayers equivalently to the treatment with CypA with PPIase activity (Fig. 1e–g), indicating that CypA-triggered loss of barrier properties of bEnd.3 cells is independent of PPIase activity. Next, we investigated whether basigin is involved in CypA treatment-induced loss of barrier properties, since CypA is known to have binding partners other than basigin. After the suppression of basigin expression by introducing the siRNAs specific for basigin into bEnd.3 cells^7^, the cells were treated with CypA. In bEnd.3 monolayers with suppressed expression of basigin, no significant decrease in the levels of cell membrane-localized claudin-5 as well as the TEERs was observed by CyPA treatment (Fig. 2). Thus, basigin was confirmed to be an essential molecule for the opening of blood–brain barrier by CypA.

### Transient and reversible loss of barrier properties with CypA treatment

**Figure 3:**
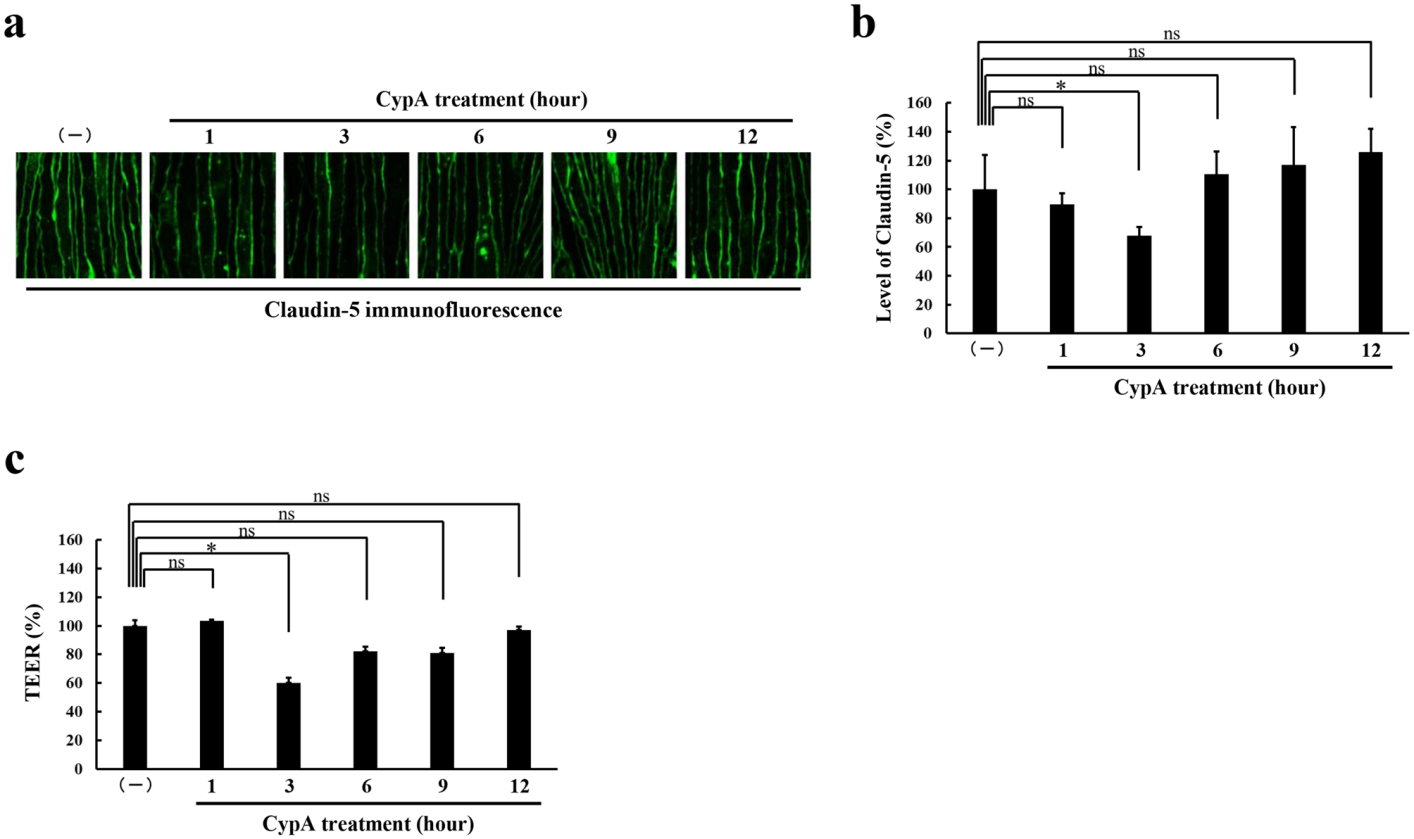
Transient and reversible loss of barrier properties in CypA-stimulated brain microvascular endothelial cells. Immunofluorescence staining (**a**) and its corresponding quantitative analysis (**b**) of cell membrane-localized claudin-5 reveal that the levels of cell membrane-localized claudin-5 are significantly decreased 3 h after the CypA stimulation and restored to the unstimulated level already 9 h after the stimulation. TEERs of monolayers (**c**) show the close correlation with the levels of cell membrane-localized claudin-5. *P < 0.05; *ns* not significant.

**Figure 4:**
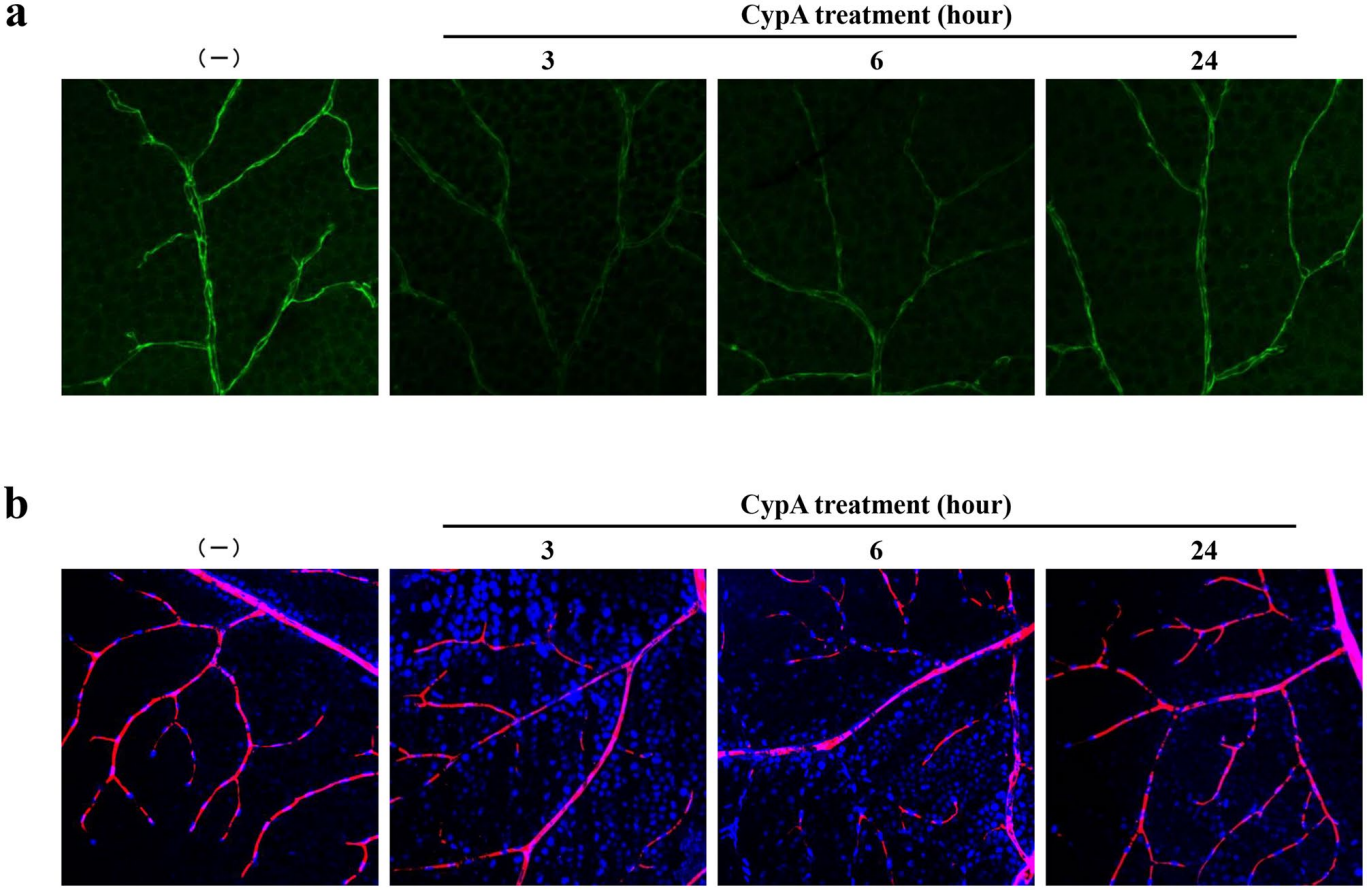
Transient and reversible loss of barrier function of retinal vasculature of mice with intravenous injection of CypA. Expression of claudin-5 (**a**) and the permeability (**b**) of retinal vasculature were determined 3, 6 and 24 h after the intravenous injection of CypA by immunofluorescence staining (**a**) and permeability assay with fluorescent dyes, Hoechst stain (blue) and dextran (red) (**b**). Significant decrease in levels of cell membrane-localized claudin-5 as well as the significant extravasation of Hoechst stain (blue) are observed 3 h after the injection of CypA. Both the levels of cell membrane-localized claudin-5 and the vascular permeability return to the physiological levels 24 h after the CypA injection.

To determine the time course of the effect of CypA teatment on bEnd.3 monolayers, the monolayers were incubated with 300 ng/ml of CypA for different periods. Decrease in the levels of cell membrane-localized claudin-5 as well as the TEERs of bEnd.3 monolayers were detectable after 3 h of CypA treatment, and returned to those of unstimulated bEnd.3 monolayers after 9 h of treatment in a self-limiting manner (Fig. 3). These interesting data indicate that the loss of barrier properties of bEnd.3 monolayers by CypA is self-limiting, transient and reversible. Subsequently, we examined whether this interesting finding is also true in vivo, considering the clinical application of CypA-triggered opening of vascular barrier for drug delivery to the parenchyma of neural tissues. We first analyzed the changes in the endothelial cell membrane-localized claudin-5 as well as the permeability of retinal vasculature, according to the methods described previously^6,7^, in mice to which a single dose of CypA was administered through their caudal veins. As shown in Fig. 4a, the levels of claudin-5 in cell membranes of peripheral microvascular endothelial cells were found to be decreased in 3 h after an intravenous injection of CypA, while they were restored to the physiological level in 24 h. In parallel with the changes in cell membrane-localized claudin-5 levels, the leakage of intracardially injected dye was enhanced in 3 h and returned to the undetectable physiological level in 24 h after an injection of CypA (Fig. 4b). Throughout the period of experiment, neither the significant brain edema, which was assessed by measuring wet and dry weight of brains (Supplementary Fig. S2), nor neurological signs were detected in mice.

### Enhanced delivery of drugs into brain parenchyma by pre-injection of CypA

**Figure 5:**
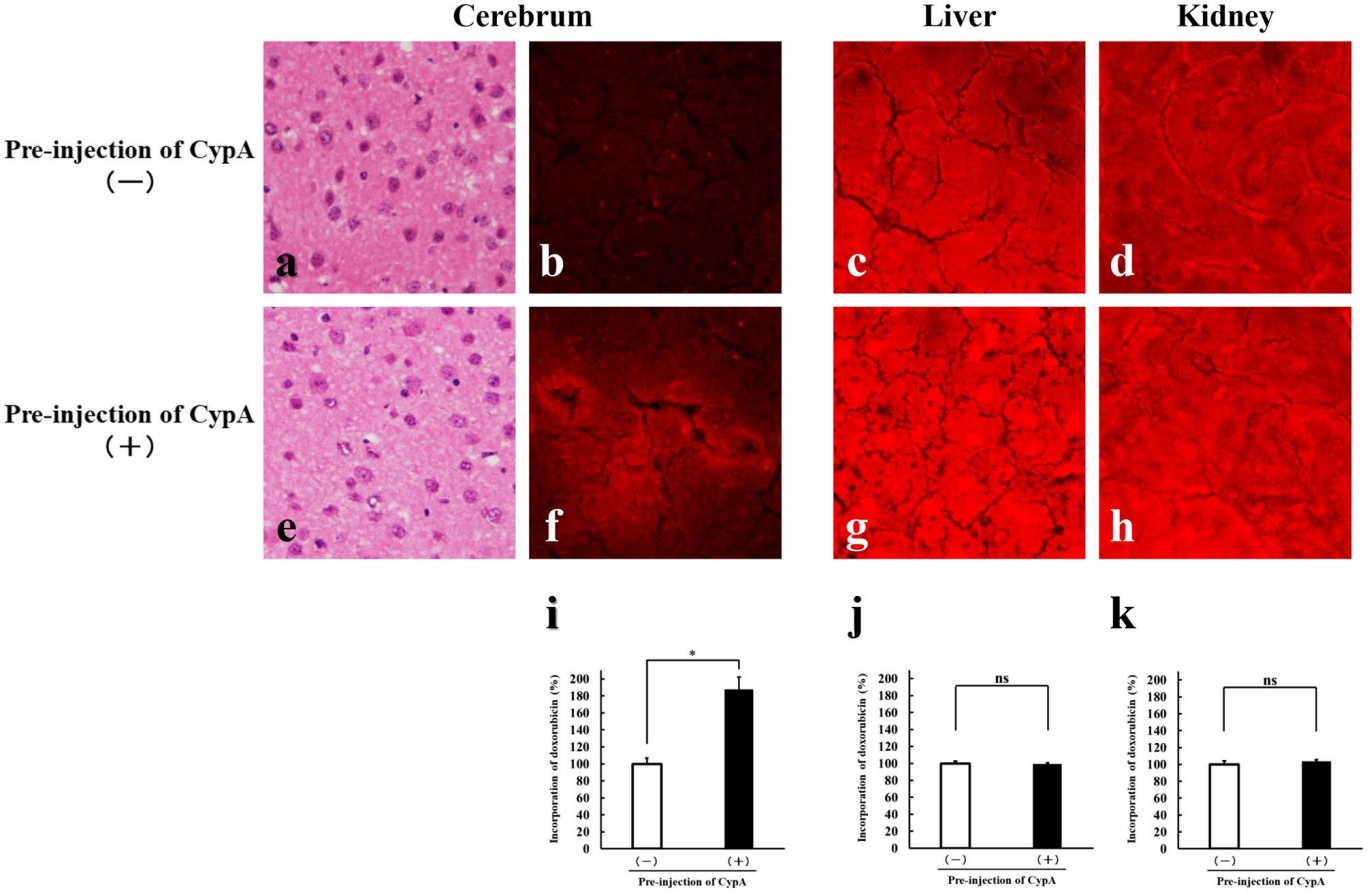
Delivery of doxorubicin into cerebral parenchyma by pre-injection of CypA. Doxorubicin was administered intravenously into mice without (**a**–**d**) or with (**e**–**h**) pre-injection of CypA. Histological analyses of cerebrum (Hematoxylin–Eosin; **a**,**e**) were performed, and the incorporation of doxorubicin into parenchyma of cerebrum, liver and kidney was visualized (red signals in **b**–**d**,**f**–**h**) as well as quantified (**i**–**k**) by taking advantage of its fluorescent nature. In mice with pre-injection of CypA, the significant enhancement of fluorescence signals of doxorubicin is observed in cerebrum (**f**), in contrast to the minimal signals in cerebrum of mice without pre-injection of CypA (**b**). As for liver (**c**,**g**) and kidney (**d**,**h**), the intense signals of doxorubicin are obtained regardless of the pre-injection of CypA. *P < 0.05; *ns* not significant.

Our present data, showing that the intravenous injection of a single dose of CypA results in the transient as well as reversible opening of vascular barrier of retinal vasculature, prompted us to discuss if the pre-injection of CypA enables therapeutic drugs to enter the parenchyma of neural tissues. We administered the doxorubicin intravenously into the mice without or with pre-injection of CypA, and its incorporation into parenchyma of cerebrum, liver and kidney was analyzed under microscope by taking advantage of its fluorescent nature. As for cerebrum, minimal fluorescence signals were detectable in mice without pre-injection of CypA (Fig. 5b), while enhanced fluorescence signals of doxorubicin were obtained in mice with pre-injection of CypA (Fig. 5f). No significant histopathological changes were detected in cerebrum between mice without and with pre-injection of CypA (Fig. 5a,e). By contrast, in liver and kidney which have the vasculature without barrier function, intense fluorescence signals of doxorubicin were detected in their parenchyma of mice without as well as with pre-injection of CypA, and the fluorescence signal intensity was not influenced significantly by the pre-injection of CypA (Fig. 5c,d,g,h). These findings were confirmed by quantitative analyses (Fig. 5i–k). These data clearly demonstrate that the pre-injection of a single dose of CypA allows doxorubicin to go into the parenchyma of neural tissues.

## Discussion

Blood–brain barrier function is not a static property, but it is dynamically regulated depending on the circumstances to which the vasculature is exposed^14,15^. Blood–brain barrier is known to be opened in various pathological conditions such as ischemic and inflammatory processes, and many studies have been reported so far regarding the mechanisms underlying the loss of barrier function in various neural diseases^16–19^. Most of those studies have been thought to be focused on the phenomena to the degree of degradation of barrier-forming molecules, including those by proteinases such as matrix metalloproteinase 9^20–22^. In our laboratory, considering that molecules involved in adaptive response would be the preferable targets for artificial regulation of blood–brain barrier function, we have focused our study rather on the mechanisms underlying the opening of blood–brain barrier which must be physiological adaptive response of endothelial cells to the changes in tissue microenvironment. Consequently, we have specified ADAM12, ADAM17 and basigin as the molecules which are expressed in barrier-forming endothelial cells and involved in the opening of blood–brain barrier in response to tissue hypoxia, cytokines and so forth^6,7^. ADAM12, ADAM17 as well as basigin are transmembrane molecules already expressed in neural vascular endothelial cells under physiological condition, and therefore they would enable the endothelial cells to change their vascular barrier function promptly without new transcription of genes for other molecules. Among these molecules, we have selected basigin as a target to establish the procedure for artificial opening of blood–brain barrier. Basigin is a transmembrane molecule of immunoglobulin superfamily which was discovered by its possible involvement in diverse physiological and pathological processes including the induction of matrix metalloproteinases, the modulation of inflammation as well as the blood–brain barrier function. Therefore, basigin has several synonyms; extracellular matrix metalloproteinase inducer (EMMPRIN), CD147, HT7 and so forth^23^. In the context of its possible involvement in blood–brain barrier function, basigin was first reported as HT7 in chick which is expressed exclusively in barrier-forming endothelial cells as for vascular endothelial cells^24^. After the discovery of basigin, its function in blood–brain barrier function had not been specified regardless of many studies including those with gene targeting. Recently, we reported that basigin expressed in barrier-forming endothelial cells is essential for the opening of blood–brain barrier, not for the maintenance of barrier, and furthermore that basigin is a candidate of the target molecule to restore the barrier function in vasculature which loses barrier function in neural diseases^7^. In the present study, we were successful in revealing another availability of basigin as a target for the artificial opening of blood–brain barrier to enhance the delivery of therapeutic drugs into the parenchyma of neural tissues. Our in vitro as well as in vivo experiments have clearly demonstrated that the blood–brain barrier can be artificially opened by CypA which is administered extracellularly to endothelial cells. Furthermore, it is noteworthy that the artificial opening of barrier by CypA is transient as well as reversible from hour to hour without significant neurological signs. Although CypA is well known as a mediator to modulate the inflammatory processes through interaction with various molecules^10,25–27^, our data with specific siRNAs for basigin demonstrated that basigin is essential for CypA-induced barrier opening. Another interesting data showing that extracellular CypA can open the barrier independent of its PPIase activity might lead to the clinical application of the CypA homologue lacking PPIase activity to reduce side effects.

Difficulty in delivering hydrophilic drugs to the parenchyma of neural tissues is one of the most serious problems to be solved for the improvement of prognosis of patients with various neural diseases^28^. In the present study, we were successful in detecting the intravenously administered doxorubicin in cerebrum of mice in which CypA was pre-injected, in contrast to its minimal incorporation into cerebrum of mice without pre-injection of CypA. However, it is important to consider that the restriction of movement of molecules between blood and tissue parenchyma by blood–brain barrier is essential to maintain the optimal tissue microenvironment for normal functioning of neural cells. In this context, it is noteworthy that the opening of blood–brain barrier by a pre-injection of CypA was shown to be transient as well as reversible by our permeability assay with tracers. Therefore, the basigin-mediated barrier opening by CypA is thought to be a promising candidate of new therapeutic strategy to improve the prognosis of patients with various intractable neural diseases by enabling the efficient delivery of drugs to cells in neural tissues.

## Methods

### Cell culture

A mouse brain microvascular endothelial cell line, bEnd.3, was obtained from American Type Culture Collection (Manassas, VA), and was grown as a monolayer at 37 °C under 5% CO_2_ in Dulbecco’s modified Eagle’s medium containing 4500 mg/l glucose (Sigma-Aldrich, St. Louis, MO, USA) supplemented with 10% fetal bovine serum. All experiments were performed with the cells at confluent state for 7 days. For treatment with tunicamycin, the cells were cultured in the presence of tunicamycin (3.75 μg/ml) for 24 h. Cyclophilin A with peptidyl-prolyl cis–trans isomerase (PPIase) activity (CypA) and Cyclophilin A without PPIase activity (CypA/PPIase-) were purchase from BioVendor research and diagnostic products, Brno, Czech Republic and Abcam, Cambridge, UK, respectively. For CypA stimlation, the cells were incubated with 200, 300 or 400 ng/ml of CypA for 1, 3, 6, 9 or 12 h.

### Western blot analysis

Cells were lysed in 100 μl of PBS containing 0.5% Triton X-100, 1% sodium dodecyl sulfate (SDS) and Halt™ Protease and Phosphatase Inhibitor Cocktail (Thermo Fisher Scientific). After addition of Laemmli sample buffer (BioRad) supplemented 5% 2-mercaptoethanol (Sigma-Aldrich), the samples were boiled. Then, aliquots of samples containing 10 μg protein were separated by SDS–polyacrylamide gel electrophoresis (SDS-PAGE) and transferred to polyvinylidene difluoride membranes, Immobilon P membranes (Millipore). After the incubation with Tris-buffered saline with 0.1% Tween 20 (TBS-T) containing 5% skim milk for blocking non-specific bindings, membranes were reacted with rabbit polyclonal antibody against basigin (1 μg/ml; generated by Scrum, Tokyo, Japan) at 4 °C overnight. After the wash with TBS-T, they were incubated with horseradish peroxidase (HRP) conjugated goat anti-rabbit IgG (1/1000 dilution; Dako) for 1h at room temperature. For standardization, the membranes were re-probed with mouse monoclonal antibody against β-actin (1/15,000 dilution; Sigma-Aldrich, Inc. MO, USA) for 15 min at room temperature. After the wash with TBS-T, they were incubated with HRP conjugated goat anti-mouse IgG (1/1000 dilution; Dako) for 15 min at room temperature. Then, they were reacted with Amersham ECL start or ECL prime (GE Healthcare, Uppsala, Sweden) according to the manufacturers’ instructions. Then chemiluminescence was detected using Amersham Imager 600 (GE Healthcare, Uppsala, Sweden).

### Immunofluorescence staining

Cultured bEnd.3 cells were fixed with 100% methanol for 5 min at room temperature, and were incubated with 10% Non-Immune Goat Serum (Invitrogen, Carlsbad, CA) for 30 min to block the non-specific binding of antibodies. Then the cells were reacted with rabbit polyclonal antibody against claudin-5 (1/25 dilution; Invitrogen, Carls bad, CA) at 4 °C overnight. After washing with phosphate-buffered saline (PBS), the cells were incubated with Alexa Fluor 488 goat anti-rabbits IgG (1/200 dilution; Molecular probes Eugene, OR) for 1 h at room temperature under protection from light. Stained cells were mounted in Fluoromount (Diagnostic BioSystems, Pleasanton, CA) and observed under a Zeiss LSM5 Pascal laser confocal microscope (Carl Zeiss, Jena, Germany). For a quantitative analysis, the fluorescence intensities of claudin-5 on plasma membranes were measured using an operation menu installed in LSM5 Pascal^6,7^. Three fields of a cultured dish were randomly photographed, and 5 straight lines were drawn on each photograph. Then, fluorescence intensities at the points of cell membranes intersected with drawn straight lines were quantified. The mean value of fluorescence intensities, at around 80 points, was calculated as the level of claudin-5 on cell membrane for each monolayer. All experiments were performed independently in triplicate.

### Transfection of small interfering RNA (siRNA)

Non-silencing siRNA for negative control and siRNAs specific for mouse basigin (ID: s63099 and s63100, defined as basigin siRNAs #1 and #2) were purchased from Applied Biosystems (Applied Biosystems, Foster City, CA). Transfection of siRNAs was performed using Lipofectamine RNAiMAX (Thermo Fisher Scientific) and Opti-MEM I (Thermo Fisher Scientific) according to the manufacturers’ instructions. Final concentration of siRNAs was 20 nM. After 48 h, the cells were processed for experiments. Specific and significant silencing of target molecules had been confirmed as described previously^7^.

### Transendothelial electrical resistance (TEER)

Electrical resistance across a bEnd.3 monolayer was measured as described previously^5–7^. In brief, bEnd.3 cells were grown on fibronectin-coated inserts of 0.4 mm pore size to the confluence, and the electrical resistance of the inserts was measured with the Millicell ERS Voltohmmeter (Millipore, Billerica, MA). TEERs of bEnd.3 monolayers were calculated by subtracting the resistance of blank inserts without cells and multiplying the subtracted values by the surface areas of inserts. Each experiment was performed in triplicate.

### Animal studies

Male C57B6J mice (7 weeks old; Japan SLC, Shizuoka, Japan) were used in this study. All experimental procedures were approved by the Institutional Animal Care and Use Committee (IACUC) in Yamaguchi University, and performed according to the Rule for the Care and Use of Laboratory Animals in Yamaguchi University and The Law (No. 105), Notification (No. 88) and Guideline (No. 71) of the Government. The reporting in the manuscript follows the recommendations in the ARRIVE guidelines.

### Immunofluorescence staining of retinal flat mounts

Flat mounts of retinas were fixed with 4% PFA for 30 min at room temperature, and were treated with PBS containing 1% BSA and 0.5% Triton X-100 for 1 h at room temperature to block the non-specific binding of antibodies as well as the permeabilization of tissues. Subsequently, the flat mounts were reacted with rabbit polyclonal antibody against claudin-5 (1/25 dilution; Invitrogen) at 4 °C overnight. After washing with PBS containing 0.1% Tween20, they were incubated with Alexa Fluor 488 goat anti-rabbit IgG (1/200 dilution; Molecular Probes) for 3 h at room temperature. The retinal flat mounts were then washed with PBS and mounted in fluorescent mounting medium for observation under a Zeiss LSM5 Pascal laser confocal microscope (Carl Zeiss).

### Permeability assay of retinal vasculature

To investigate the effect of CypA on blood–brain barrier function in vivo, CypA or its vehicle for negative control was injected intravenously once into mice (200 μg/kg), and the permeability of retinal vasculature was determined 3, 6 and 24 h after the injection of CypA as described previously^5–7^. Briefly, 500 μl of PBS containing 100 μg/ml Hoechst stain H33258 (molecular mass, 534 Da; Sigma-Aldrich) and 1 mg/ml tetramethylrhodamine-conjugated lysine-fixable dextran (molecular mass, 10,000 Da; Thermo Fisher Scientific) were injected into the left ventricle. After the injection of fluorescent dyes, eyes were enucleated and immediately fixed in 4% paraformaldehyde (PFA) for 15 min at room temperature under protection from light, and retinal flat mounts were prepared. They were mounted in fluorescent mounting medium and observed under a Zeiss LSM510 META laser confocal microscope (Carl Zeiss). Experiments were performed independently at least 3 times.

### Assessment of brain edma

For the assessment of brain edema formation by the opening of blood–brain barrier with CypA treatment, the water content of brains of mice 3 and 24 h after the injection of CypA as well as control mice is quantified by measuring wet and dry weight of brains. To measure the dry weight, water of brains was evaporated at 110 °C for 72 h. The percentage of water content in a brain was calculated as 100 × (wet weight − dry weight)/wet weight, and compared between the control mice and the mice with CypA treatment.

### Detection of intravenously injected doxorubicin

Doxorubicin Hydrochloride (6.25 mg/kg; FUJIFILM Wako Pure Chemical Corporaion, Osaka, Japan) was injected into caudal veins of mice without or with pre-injection of CypA (200 μg/kg). Pre-injection of CypA or its vehicle for negative control was performed intravenously 3 h prior to the injection of doxorubicin. Three hours later, the mice were sacrificed, and cerebrum, liver and kidney were collected and frozen in optimal cutting temperature (OCT) compound. Then, the frozen sections of 10 μm thickness were prepared and observed under an LSM710 laser confocal microscope (Carl Zeiss, Jena, Germany). Fluorescence of doxorubicin was excited with an argon laser at 488 nm, and the emission was observed through a 530-nm long-pass filter^29,30^. For quantitative analysis, three sections were prepared from a cerebrum, liver or kidney, and three areas per section were randomly selected under 20× objective lens. Fluorescence intensity of each area was quantified with an operation menu installed in LSM710 laser confocal microscope, and then the average of 9 areas was calculated for a sample. Three independent experiments were performed.

### Statistical analysis

All data are presented as means ± SD. Data were compared with Student’s t tests, since the variance was shown to be equal with F test between the groups which were to be compared in this study. Differences were considered to be statistically significant at *p* < 0.05.

## Supplementary Information

Supplementary Information.

## Data Availability

Data are available from the corresponding author upon request.
