# Supplementary material for: Intravenous injection of cyclophilin A realizes the transient and reversible opening of barrier of neural vasculature through basigin in endothelial cells
Source: Sci Rep. 2021 Sep 29;11:19391. doi: 10.1038/s41598-021-98163-w (PMC8481259; doi:10.1038/s41598-021-98163-w)
Supplement: Supplementary file 1 — Supplementary Information. [file 41598_2021_98163_MOESM1_ESM.pdf]

Supplementary information

Intravenous injection of cyclophilin A realizes the transient and reversible opening of barrier of neural vasculature through basigin in endothelial cells

Narumi Nakada-Honda, Dan Cui, Satoshi Matsuda, Eiji Ikeda

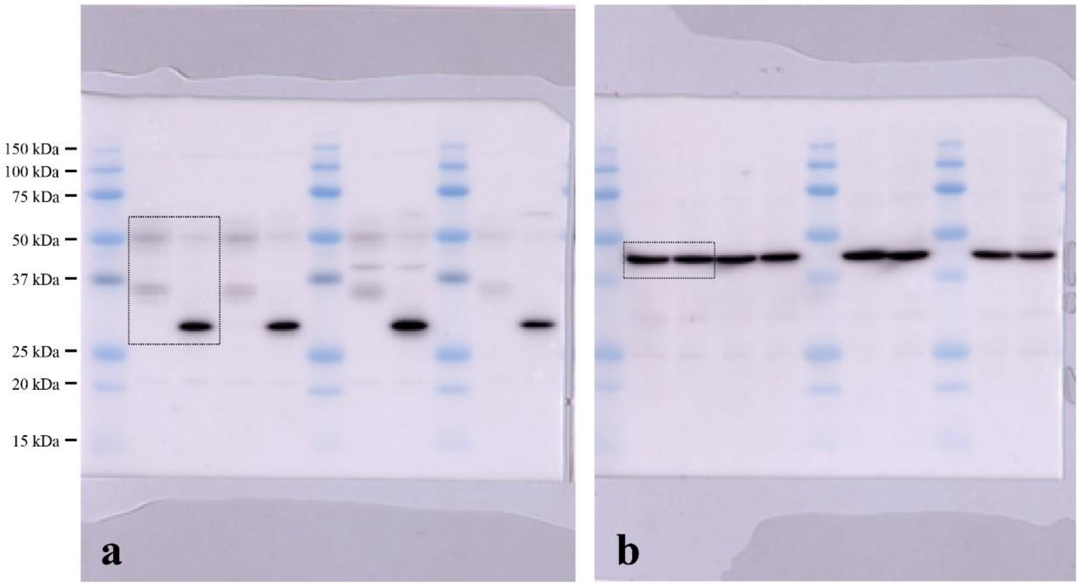

Supplementary Figure S1: The images of full-length membranes for Figure 1a. Parts of images (a, basigin; b, β-actin) surrounded by squares were processed for Figure 1a.

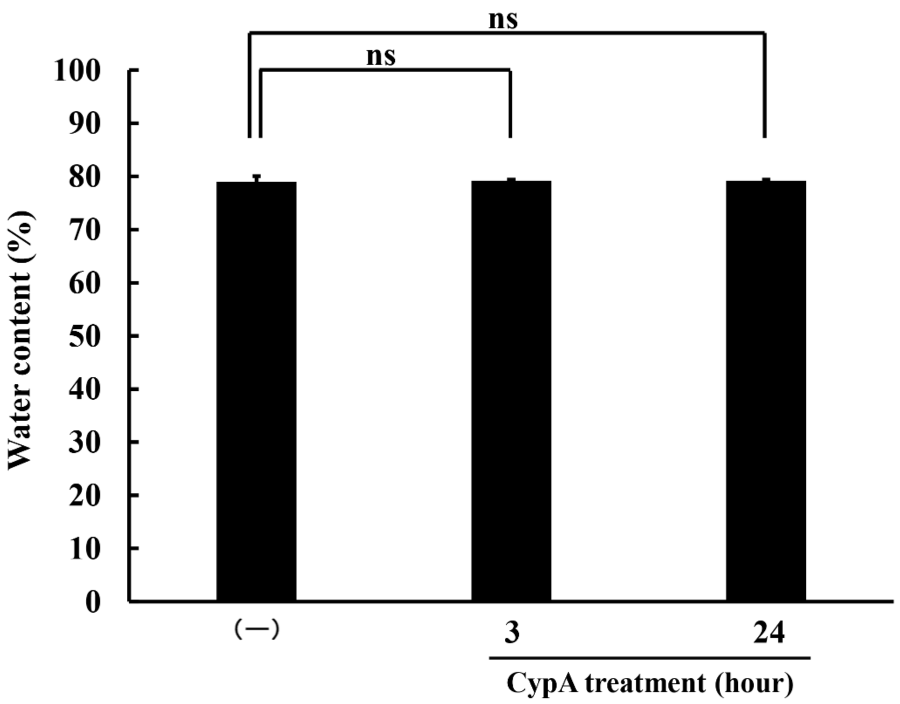

Supplementary Figure S2: The water content of brains of mice after the injection of CypA. Wet weight and dry weight of brains of mice without as well as with the injection of CypA were measured to evaluate the edema formation. No significant increase in the water content of brain, which was calculated as 100 x (wet weight – dry weight)/wet weight, is detected 3 and 24 hours after the injection of CypA. Data are presented as mean ± SD from 3 independent experiments. ns, not significant.
